# Visualizing the Role of 2’-OH rRNA Methylations in the Human Ribosome Structure

**DOI:** 10.3390/biom8040125

**Published:** 2018-10-25

**Authors:** S. Kundhavai Natchiar, Alexander G. Myasnikov, Isabelle Hazemann, Bruno P. Klaholz

**Affiliations:** 1 Centre for Integrative Biology (CBI), Department of Integrated Structural Biology, IGBMC, CNRS, Inserm, Université de Strasbourg, 1 rue Laurent Fries, 67404 Illkirch, France; natchiar@igbmc.fr (S.K.N.); myasniko@igbmc.fr (A.G.M.); hazemann@igbmc.fr (I.H.); 2 Institute of Genetics and of Molecular and Cellular Biology (IGBMC), 1 rue Laurent Fries, 67404 Illkirch, France; 3 Centre National de la Recherche Scientifique (CNRS), UMR 7104, 67404 Illkirch, France; 4 Institut National de la Santé et de la Recherche Médicale (Inserm), U964, 67404 Illkirch, France; 5 Faculté des Sciences de la Vie, Université de Strasbourg, 67404 Illkirch, France

**Keywords:** RNA, chemical modifications, ribosomal RNA, 2′-*O* methylation, human ribosome, structural biology

## Abstract

Chemical modifications of RNA have recently gained new attention in biological sciences. They occur notably on messenger RNA (mRNA) and ribosomal RNA (rRNA) and are important for various cellular functions, but their molecular mechanism of action is yet to be understood in detail. Ribosomes are large ribonucleoprotein assemblies, which synthesize proteins in all organisms. Human ribosomes, for example, carry more than 200 modified nucleotides, which are introduced during biogenesis. Chemically modified nucleotides may appear to be only scarcely different from canonical nucleotides, but modifications such as methylations can in fact modulate their chemical and topological properties in the RNA and alter or modulate the overall translation efficiency of the ribosomes resulting in dysfunction of the translation machinery. Recent functional analysis and high-resolution ribosome structures have revealed a large repertoire of modification sites comprising different modification types. In this review, we focus on 2′-*O*-methylations (2′-*O*-Me) and discuss the structural insights gained through our recent cryo electron microscopy (cryo-EM) high-resolution structural analysis of the human ribosome, such as their locations and their influence on the secondary and tertiary structures of human rRNAs. The detailed analysis presented here reveals that ribose conformations of the rRNA backbone differ when the 2′-OH hydroxyl position is methylated, with 3′-*endo* conformations being the default and the 2′-*endo* conformations being characteristic in that the associated base is flipped-out. We compare currently known 2′-*O*-Me sites in human rRNAs evaluated using RiboMethSeq and cryo-EM structural analysis and discuss their involvement in several human diseases.

## 1. Introduction

**Figure 1:**
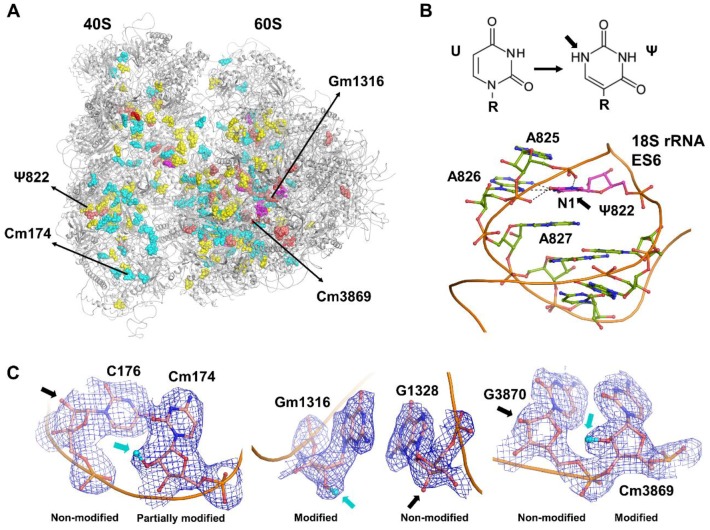
(**A**) Overview of the human 80S ribosome structure with rRNA modifications that have been described structurally. Fully methylated 2′-OH hydroxyl sites (cyan) in the human 80S ribosome structure [13] (PDB ID: 6EK0); partially methylated 2′-OH sites (red); new 2′-*O*-Me sites identified (magenta); pseudo-uridines Ψ (yellow); and base modifications (salmon). (**B**) Example of pseudo-uridine (magenta) with an additional hydrogen bond in the N1 atomic position. (**C**) Examples of modified residues (atomic model and cryo-EM (cryo electron microscopy) map in blue, neighboring non-modified nucleotides are shown for comparison): left, partially methylated Cm174 in the 18S rRNA; middle, fully methylated Gm1316 in 28S rRNA; right, methylated Cm3869 in 28S rRNA that are found to be fractionally methylated sites by RiboMethSeq analysis.

Protein synthesis is one of the fundamental cellular processes and it is performed by ribosomes, which are complex nucleoprotein nanomachineries [1,2,3,4,5,6]. Over the course of evolution ribosomes across all species preserved their overall architecture, assembly, and the composition of the main catalytic sites. In eukaryotes, during evolution the size of the ribosomes increased substantially as did the number of ribosomal proteins and nucleotides in the ribosomal RNAs (rRNAs) [7,8,9,10,11], with the exception of archaea in which evolution also occurred by partial loss [12]. In human cytosolic 80S ribosomes, the large subunit comprises of 28S, 5.8S, and 5S rRNAs and 47 ribosomal proteins and the small subunit contains 18S rRNA and 33 ribosomal proteins [11,13]. However, while ribosomes could be thought of being identical within a given species, heterogeneous ribosome populations in cells can occur during biogenesis as a result of stage-specific expression of rDNA genes, activation of cell-specific genes, alterations in pre-rRNA processing, and differential post-transcription and post-translation modifications [14,15,16]. Eventually, these processes locally alter the chemical composition of ribosomal proteins and nucleotides with various site-specific chemical modifications. More than 200 nucleotides were found biochemically to be modified in human ribosomes. Those chemical modifications in rRNAs are diverse in nature and include ribose 2′-OH hydroxyl methylations, isomerizations of uridines to pseudo-uridines (Ψ), and modifications at various atomic positions of the cyclic nucleotide bases including methylations and different types of hypermodifications [17,18]. Unlike in prokaryotic ribosomes where 2′-*O*-Me sites represent only a minority of the modification sites (*Escherichia coli*: 9% and *Thermus thermophilus*: 17% of the modified sites), they are very abundant in eukaryotic ribosomes (Figure 1A). For example, 51% and 48% of the modified nucleotides in human and yeast ribosomes, respectively, are 2′-OH methylations. Other modifications are base modifications of which more than 40% are pseudo-uridines (Ψ’s) and only less than 10% of the modification sites are located directly at the cyclic bases [17].

Chemical modifications of the rRNAs are epitranscriptomic changes introduced by numerous enzymes and ribonucleoprotein and small nucleolar RNA (snoRNA) complexes at different stages of ribosomal subunit maturation and are important for fine-tuning the structure and function of the ribosomes [19,20]. Chemical modifications appear to be one of the sources of ribosome heterogeneity in relation to location, chemical composition, and the level of occurrence, the current working hypothesis being that “specialized ribosomes” may exist in response to distinct cellular activities. In some cases, the plasticity of rRNA modifications can provide functional specificity to the ribosomes, which influences the ribosome fidelity to translate messenger RNAs (mRNAs) [21,22] and in several cases correlate with specific ribosomopathy diseases [23,24]. Hence, even though for the vast majority of rRNA modifications the role and implications are still unknown, several studies have revealed that altered ribose methylation levels in rRNAs generate heterogeneous subpopulations of ribosomes that may affect normal and pathophysiological processes in human cells. It should also be noted that chemical modifications of the mRNA such as *N6*-methyladenosine (m^6^A) are involved in translation regulation of the associated genes [25,26,27,28]. In this review we discuss the structural and functional implications of rRNA modifications in ribosomes with a specific focus on 2′-*O*-methylation in human cytoplasmic ribosomes and the differences observed for 2′-*endo* and 3′-*endo* ribose conformations.

## 2. Locations and Functional Implications

In the past, many chemical modifications of the rRNAs have been identified biochemically or with chemistry-assisted tools and, in particular, through sequencing approaches (e.g., RNAseq, RiboMethSeq [29], MeRIP-seq for m^6^A [30], and Aza-RIP for m^5^C [31,32]; reverse-transcription based methods for 2′-*O*-Me [33], ligation-based methods for 2′-*O*-Me, Ψ, and m^6^A [34,35] and *pseudo*-seq for Ψ [36]) and they have been listed in databases such as the three-dimensional (3D) rRNA modification maps database [17], RNAMDB [19], MODOMICS [18,37], and MeT-DB [38]. However, their structural environment within the ribosome and their role therein was unknown until ribosome structures were recently resolved to a resolution where chemical modifications can be directly observed. To date, rRNA modifications have been structurally visualized in ribosomes from *E. coli* [39,40], *T. thermophilus* [41], *Leshmania donovani* [42], and human [13] and the number of chemical modifications occurring in rRNAs is ranging from a few tens in prokaryotes to more than a hundred in eukaryotes [19]. In higher eukaryotes, the evolutionary complexity of rRNAs and ribosomal proteins is further extended by the amount of modifications and their location in the ribosomes [13,42,43] suggesting the existence of an extended shell of modifications in eukaryotic and human ribosomes [13]. On the basis of our recent high-resolution structural analysis of the human ribosome [13] we classified the modification sites as universally conserved locations (class I), human or eukaryote-specific modifications (class II), and some new unpredicted sites (class III) that remain to be further characterized to address their chemical nature; the suggested new class III sites were addressed based on the occurrence of an additional density as compared to their nonmodified nearest neighbor nucleotides (i.e., similar to difference mapping) and their presence needs to be confirmed biochemically. We will discuss some of the rRNA modifications below to highlight their structural roles, with a focus on 2′-*O*-methylations which are numerous and for which more functional data are available as well. Most of the ~10 modified nucleotides that cluster at the various functional sites of the ribosomes in prokaryotes are carried forward to eukaryotes, which are crucial for the assembly, structure, and function of ribosomes [44,45]. In the human ribosome (Figure 1), human or eukaryote-specific sites comprise predominantly 2′-OH methylations and pseudo-uridylations (Figure 1A) that are also located at the functional sites of the ribosome (e.g., peptidyl transferase center (PTC) and decoding center (DC)). In general, unless specific hydrogen-bonding patterns are visible in the structure, pseudo-uridines cannot be identified in the structure due to their isomeric nature (thus among the ~100 predicted Ψs only 21 could be described).

The structural analysis of the human ribosome and its comparison with the structure of other ribosomes [13,39,40,41,42,46,47] reveals that modified nucleotides are mostly found in various loops in the rRNAs and in bulges in the middle of long rRNA helices, thereby maintaining the structural motifs of the rRNA structure. For example, Ψs, which provide additional hydrogen (*H*) bond potential, are mostly located at the termini of rRNA helices, in rRNA loops and in regions that require more stability for the fold of the rRNA tertiary structure (Figure 1B). Furthermore, crucial catalytic activities of protein biosynthesis occur in domains IV, V, and VI in the large ribosomal subunit, which are amended with chemical modifications [44]. Domain V possesses the most important catalytic site: the PTC, where the helices and multijunction loops that embrace the PTC are rich in chemical modifications. Especially helix H89 (inter-subunit bridge and transfer RNA (tRNA) peptidyl (P) site) is exceptionally furnished with Ψs. Similarly, the amino acyl (A) and P tRNA binding sites are stabilized with five Ψs. On the one hand, domains II and III are mostly decorated with 2′-*O*-Me sites, on the other hand helices H37, H38, and H39 (A-site finger) are mostly furnished with Ψs with the exception of Am1871. On the small subunit, modified nucleotides are clustered mostly at the 5’ and central domains, where large portions of these domains comprise the important functional sites, the decoding center and the A-, P-, and exit (E)-sites of the tRNAs, which comprise the anticodon stem binding regions. Although for many sites there are no functional data yet, for some sites their functional role has been characterized. For example, loss of modifications at the P-site impairs subunit assembly, and a eukaryotic conserved hypermodification (aminocarboxypropyl, acp) m^1^acp^3^ψ1248 in the 18S rRNA at the P-site influences the subunit assembly [48]. In addition, two dimethyl adenines (m^6^_2_A1850 and m^6^_2_A1851) are the two most universally conserved base modifications located at the inter-subunit bridge (B2a) and in vicinity of the P-site and mRNA channel [13]. This specific organization facilitates the subunit interactions near the A- and P-sites of the ribosome.

## 3. 2′-*O*-Me in Human 80S rRNAs: 2′-*Endo* vs. 3′-*Endo* Conformations

**Figure 2:**
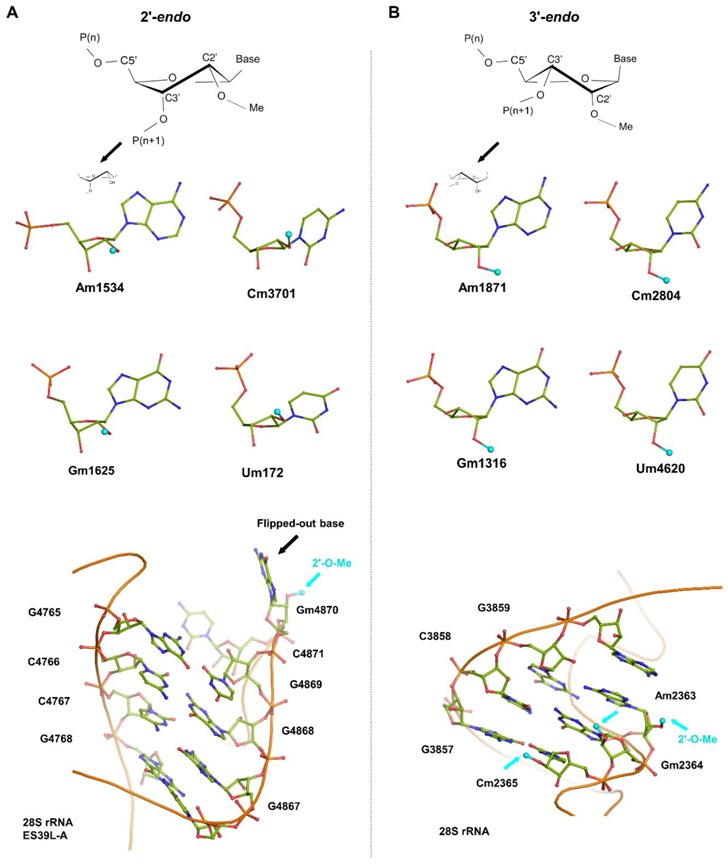
The analysis of 2′-*O*-methylation sites in the human ribosome structure reveals the presence of two different ribose conformations (2′-*endo* and 3′-*endo*). Examples are given for each of the A, C, G, and U nucleotide types. (**A**) Examples of 2′-*endo* nucleotides and (bottom) a characteristic base flip-out at the edge of an rRNA helix. (**B**) Examples of 3′-*endo* nucleotides; the 2′-*O*-Me moiety is in plane with the nucleotide base, which allows extending base stacking between neighboring bases (bottom).

Ribose methylation is one of the most abundant modifications occurring in human 80S ribosomes (Figure 1C and Figure 2). It has been shown to be crucial for mRNA selection and translation fidelity [22,45,49]. 2′-OH ribose methylations (and also Ψs) are essential for maintaining the structure and function of the rRNA [13]. In general, rRNA’s possess two different ribose conformations: 2′-*endo* (Figure 2A) and 3′-*endo* (Figure 2B). Interestingly, we find that rRNA helices are predominantly composed of ribonucleotides that adopt a C3′-*endo* conformation of the ribose moiety (Figure 2B). The 3′-*endo* conformation implies that the furanose (pentose) ring adopts a conformation in which the free 2′-OH group is more exposed than in the 2′-*endo* conformation, and the base is positioned at a steeper angle (Figure 2A) [13,39,40,41,42]. Therefore, 2′-*O*-Me at the 3′-*endo* ribose moiety will extend the planarity of the base to enhance stacking interactions with the neighboring bases, where the 2′-*O*-Me increases the intra-nucleotide stability; this augments the conformational rigidity in the bulges and flexible secondary structures found in the middle of long rRNA helices. Hence, in 3′-*endo* ribonucleotides, the steric repulsion between 3′-OH and 2′-OH groups on a ribose reorients the 2′-*O*-Me moiety parallel to the direction of the N1-C2 bond of the heterocyclic bases of pyrimidine and purine nucleotides, respectively (Figure 2B). This stabilizes the cyclic base and enhances the base-pairing with the complementary bases and stacking interactions with neighbor bases [13,50]. Thus, 2′-*O*-Me increases the stability of the nucleotide conformation. By contrast, 2′-*endo* ribonucleotides are found mostly at kinks and hairpins between rRNA helices. A characteristic feature of 2′-*endo* nucleotides is that the base can be found flipped out, which also avoids the 2′-*O*-Me group from interfering sterically with the base (Figure 2B).

## 4. Dynamic and Substoichiometric 2′-*O*-Me Sites in the Human Ribosome

Alterations of 2′-*O*-Me sites are one of the emerging research interests in the area of rRNA modifications because they can influence cell-specific ribosome subpopulations and the intrinsic capability of ribosomal activity. Recent studies have established complete profiles for ribose methylation and examined the alteration in 2′-*O*-Me sites in HeLa and HCT116 cell lines using RiboMethSeq [43,51] (even though the stoichiometry of some sites differs in these studies). These studies revealed the occurrence of 106 2′-*O*-Me sites, but their fractional occurrence (i.e., stoichiometry) varies between different cell types. Our recent work on the human ribosomes extracted from the HeLa cancer cell line [13] provides structural evidence and describes the 3D environment of several conserved and fractionally methylated 2′-OH sites, which have been also identified biochemically in HeLa and HCT116 cells [22,43,51]. Our high-resolution cryo-EM (cryo electron microscopy) structure visualizes 72 out of the 106 predicted RiboMethSeq sites in human 80S ribosomes, which are different with respect to their stoichiometric level (in some cases the reason for not seeing a modification in the structure can be that a given region in the structure is at lower local resolution or partially disordered and therefore not visible even though possibly present). RiboMethSeq analysis on HeLa cell ribosomes revealed seven fractionally methylated sites [22,51], which is consistent with the modifications found in HCT116 cells [43]. In addition, either 3 or 11 additional nucleotides are hypomethylated in HCT116 cells depending on the presence and absence of the tumor suppressor protein p53, respectively [22,43,51,52]. The existence of sub-stoichiometrically methylated sites is also found in the human 80S ribosome structure according to some variability in the cryo-EM map (partial sites can be identified according to the weak additional density on a given residue as compared to a non-modified neighbor residue; most of these partial sites were not listed in the structure in order to remain conservative until they can be confirmed biochemically). For example, Cm174 is found to be fractionally methylated in both cryo-EM (Figure 1C) and RiboMethSeq analysis. The structure displays good density distribution also for other sites that are partially methylated according to RiboMethSeq analysis. Two independent methods (RiboMethSeq and cryo-EM) confirm the variable degree of methylation in rRNAs (MethScore < 0.75 in RiboMethSeq; local density in the cryo-EM map; Figure 1C), which possibly results in inconsistency of only two fractionally methylated sites out of 106 (Gm1316 and Cm3869 in 28S rRNA). Nevertheless, Gm1316 and Cm3869 (28S rRNA) have MethScores above 0.5, which probably would result in more than 50% of the ribosome population being furnished with the same methylation pattern, a value that would provide a reasonably homogeneous set of ribosomes for cryo-EM structural analysis (Figure 1C). Even though particle sorting procedures are nowadays used in cryo-EM image processing to separate the main structural and conformational states of the ribosome [53,54,55] (e.g., ratcheting, pre and post-translocation states, etc.) these approaches currently do not allow to sort out heterogeneity at the residue level; thus, partially modified sites show a weaker additional density as compared to fully modified sites, but still significantly stronger than completely unmodified sites (Figure 1C). The cryo-EM structure of the human 80S ribosome exhibits several new 2′-*O*-Me sites, which are not addressed with box C/D snoRNAs) in RiboMethSeq analysis [22,43,51]. A reason for that could be that the sites are not accessible in the rRNA, or that perhaps their methylation rate is too low to be identified using RiboMethSeq (in fact, some earlier reported sites could not be confirmed by RiboMetSeq). Downregulation of fibrillarin (FBL) induces a global and site-specific reduction of 2′-*O*-Me sites. Fibrillarin is an rRNA methyl-transferase that alters the overall methylation pattern in rRNA [56]. In HeLa ribosomes, FBL depletion shows less influence on 18S rRNA, whereas A- and P-sites, inter-subunit bridges and the peptide exit tunnel are considerably reduced in their methylation amount. Reversely, several 2′-*O*-Me sites at the PTC, Gm4166 in the P-site and Gm4469 in the A-site tRNA binding sites are unaffected by FBL [22]. Similarly, HCT116 cells lines in the absence of p53 also showed an increased number of undermethylated sites. Seven out of 16 fractionally modified sites are consistent with isogenic diploid HCT116 cell lines with and without p53 [43]. Taken together, the majority of the human-specific invariant 2′-*O*-Me sites are fully methylated, which are seen in the human 80S ribosome structure to be mostly involved in rRNA folding. In addition, 1/3 of the 2′-*O*-Me sites that are fractionally modified depict plasticity in 2′-*O*-Me sites, which supports the existence of specialized ribosomes in cell lines and tissues [22,43,51].

Ribosome production, composition, and intrinsic ribosome activity during translation are strongly associated with cell growth, which control the expression level of several tumor suppressors and proto-oncogenes [33,38,57,58]. Even though the precise functional role of rRNA modifications is not yet elucidated, several human diseases strongly correlate with the variations in occurrence of the rRNA modification patterns in human ribosomes. In aggressive breast cancer cells, increased tumorigenicity is associated with an increased rate of ribosome biogenesis, which is accompanied by the enhancement of ribose methylation in 28S and 18S rRNAs. For example, reduction in adenosine diphosphate (ADP) ribosylation factor like 2 (Arl2) alters the methylation patterns in 28S and 18S rRNAs, that specifically enhances the methylation of 28S rRNA residues (401 (390), 1625 (1612), 1871 (1858), 2861 (2848), 4228 (4198), and 4466 (4436); numbers according to the old nomenclatures are given in brackets; in the deposited ribosome structure the new nomenclature is used) and 18S rRNA (37, 1489, 1713, and 1803). The methylation frequency is six-fold higher for the nucleotide position 1803 in 18S rRNA and two-fold higher for 1858 in 28S rRNA in cancer cells [49]. Inactivation of p53 in human breast cells evidences tumorigenesis with elevated protein synthesis by increasing the expression level of fibrillarin [56]. A 2′-*O*-Me site conserved in mammals (Um14 in the 5.8S rRNA) stabilizes the secondary and tertiary structures at the junction of the 5.8S rRNA and in domain I of the 28S rRNA [59], whose low methylation level has been correlated with cell differentiation in newly synthesized rapidly growing neoplastic tissues [59,60]. In HeLa cells, our ribosome structure also suggests the occurrence of a sub-stoichiometric modification level of Um14 (5.8S rRNA) [13]. This suggests that specifically introduced chemical modifications generate heterogeneity in rRNAs that could result in more specialized cellular activities, providing an opportunity to specifically target dysregulated states in diseases. Together, these examples provide evidence for unique circumstances where one or more chemical modifications are specifically introduced into rRNAs. Perturbations in relevant cellular functions appears to have a strong correlation with cancerous cell growth, but it remains to be addressed whether these are also causative. Furthermore, the precise role of the specifically modified nucleotides is insufficiently known in relation to protein biosynthesis and diseases, so that detailed future studies are required to understand the molecular basis of impaired cellular activities and the degenerated nature of cancer cells.

## 5. Conclusions and Future Orientations

Taken together, the analysis of 2′-*O*-Me sites in rRNA has opened a new field of research providing many new unexpected insights. Biochemical identification techniques such as RiboMethSeq and high-resolution structural analysis of ribosomes with the advent of high-resolution single particle cryo-EM [61] are highly complementary approaches to address the structure and function of chemical modifications. While the structural analysis is not meant to replace sequencing methods it has the unique possibility of analyzing the 3D environment in which they are located to address in detail which interactions they form in the ribosome structure. In the future this type of analysis should include the more detailed investigation of specific sites relevant for diseases such as cancer. It will also require analyzing other modifications such as acp, Me, or acetylations of the nucleotide bases and find appropriate biochemical techniques or additional structural methods to identify the chemical nature of some of the new unknown sites for which direct biochemical evidence is not available yet. Obviously, a complication in such an analysis is the occurrence of partially modified sites, typically for 2′-*O*-Me sites, which can differ in different cell lines and between different biochemical methods used, but this is where the future challenge lies for biomedical relevant site analysis. In this respect it is normal that differences exist between different cell lines or between different biochemical or structural techniques used as reported recently [62] (published when this review was under final revision). However, they key point is that RiboMethSeq, quantitative mass spectrometry, and structural methods can give complementary information to understand the structural and functional implications of chemical modifications. One particular aspect of interest for future studies will be to also study the influence of modifications on inhibitor binding, considering that chemical modifications are found in typical ligand binding pockets of the human ribosome [13] and have been shown to influence inhibitor activity [63,64,65,66]. Such drug analysis will benefit from the capability of current high-resolution cryo-EM to visualize inhibitors in ligand binding pockets of the human ribosome [13,67].
